# Drug Repurposing for Schistosomiasis: Combinations of Drugs or Biomolecules

**DOI:** 10.3390/ph11010015

**Published:** 2018-02-05

**Authors:** Maria João Gouveia, Paul J. Brindley, Fátima Gärtner, José M. Correia da Costa, Nuno Vale

**Affiliations:** 1 UCBIO/REQUIMTE, Laboratory of Pharmacology, Department of Drug Sciences, Faculty of Pharmacy, University of Porto, Rua de Jorge Viterbo, 228, 4050-313 Porto, Portugal; mariajoaogouveia@gmail.com; 2 Center for the Study of Animal Science, ICETA, University of Porto, Praça Gomes Teixeira, Apartado 55142, 4031-401 Porto, Portugal; jose.costa@insa.min-saude.pt; 3 Department of Molecular Pathology and Immunology, Institute of Biomedical Sciences Abel Salazar (ICBAS), University of Porto, Rua de Jorge Viterbo Ferreira 228, 4050-313 Porto, Portugal; fgartner@ipatimup.pt; 4 Department of Microbiology, Immunology & Tropical Medicine, and Research Center for Neglected Diseases of Poverty, School of Medicine & Health Sciences, George Washington University, Washington, DC 20037, USA; pbrindley@email.gwu.edu; 5 Institute of Molecular Pathology and Immunology of the University of Porto (IPATIMUP), Rua Júlio Amaral de Carvalho, 45, 4200-135 Porto, Portugal; 6 Institute of Investigation and Innovation in Health (i3s), Rua Alfredo Allen, 4200-135 Porto, Portugal; 7 Department of Infectious Diseases, INSA-National Health Institute Dr. Ricardo Jorge, Rua Alexandre Herculano 321, 4000-055 Porto, Portugal

**Keywords:** schistosomiasis, drug repurposing, praziquantel, antioxidants

## Abstract

Schistosomiasis is a major neglected tropical disease. Control of schistosomiasis currently relies on a single drug, praziquantel, and despite its efficacy against the all schistosome species that parasitize humans, it displays some problematic drawbacks and alone is ineffective in counteracting adverse pathologies associated with infection. Moreover, due to the development of the potential emergence of PZQ-resistant strains, the search for additional or alternative antischistosomal drugs have become a public health priority. The current drug discovery for schistosomiasis has been slow and uninspiring. By contrast, repurposing of existing approved drugs may offer a safe, rapid and cost-effective alternative. Combined treatment with PZQ and other drugs with different mode of action, i.e., antimalarials, shows promise results. In addition, a combination of anthelminthic drugs with antioxidant might be advantageous for modulating oxidative processes associated with schistosomiasis. Herein, we review studies dealing with combination therapies that involve PZQ and other anthelminthic drugs and/or antioxidant agents in treatment of schistosomiasis. Whereas PZQ combined with antioxidant agents might or might not interfere with anthelminthic efficacy, combinations may nonetheless ameliorate tissue damage and infection-associated complications. In fact, alone or combine with other drugs, antioxidants might be a valuable adjuvant to reduce morbidity and mortality of schistosomiasis. Therefore, attempting new combinations of anthelmintic drugs with other biomolecules such as antioxidants provides new avenues for discovery of alternatives to PZQ.

## 1. Introduction

The Tropical Diseases Research arm of the World Health Organization classifies schistosomiasis as one of the major neglected tropical diseases. It is considered the most important of the helminthoses of humanity in terms of mortality and morbidity [1,2]. Schistosomiasis is endemic in 70 countries worldwide, affecting >250 million people [3,4,5]; yet this number likely is an underestimate, with recent studies documenting infection of 391–597 million people, with 800 million, mostly children, at risk of infection [6,7], and people are infected during domestic, recreational and occupational water [8,9]. 

The infectious agents of human schistosomiasis include three major species; *Schistosoma mansoni* and *S. japonicum* which cause hepato-intestinal schistosomiasis in Africa, the Middle East, South America and the Caribbean whereas *S. haematobium*, endemic in Africa and the Middle East and is responsible for urogenital schistosomiasis (UGS) [10,11]. Infection with *S. haematobium* also is classified as a group I carcinogen; UGS often leads to squamous cell carcinoma of the bladder [12]. Although schistosomiasis is generally restricted to the tropics and sub-tropics, a recent outbreak in Corsica demonstrates the potential for reemergence of this infectious disease in new, economically developed regions in southern Europe [13].

Infection follows exposure to freshwater containing free-swimming larval forms of the parasite (this larva is termed the cercaria). Cercariae penetrate intact human skin, where the larva sheds its tail and which the larva, now termed the schistosomulum, enters the bloodstream which travels via pulmonary artery to the lungs. After exiting the lungs, schistosomula re-enter the venous circulation and circulates for several weeks, until the adult schistosomes take up residence within the mesenteric veins (*S. mansoni* and *S. japonicum*) or the vesical plexus and veins that drain the ureter and nearby pelvic organs (*S. haematobium*) [3] where the worms mate, and commence egg laying about 5–7 weeks post-infection [3,14,15]. Schistosomes are long lived in these sites, often for decades, and they shed large numbers of the eggs each day. The eggs must transverse the walls of the blood vessels with the goal of reaching the lumen of the intestine or bladder to be excreted or evacuated in urine or feces, respectively [3]. However, many eggs become entrapped in the tissues and organs including the walls of the bladder, bowel, and in the liver which comes with blood of return to this organ [14,15]. The developmental cycle of the parasite is completed when eggs reach freshwater, hatch, and release the miracidium, a ciliated larva, which seeks out the aquatic snail. Following infection of the snail, the miracidium transforms into the sporocyst stage of the schistosome. The cercariae develop within the two generation of sporocyst (I and II), and are eventually released from the snail into the water, completing the developmental cycle [3]. 

Control strategies employ approaches to block transmission and reduce the disease burden including mass and targeted chemotherapy, and absence of safe water and sanitation facilities, modification of the environment, and use of molluscides [3]. The goal of these approaches includes mitigating the burden of disease, by reducing morbidity [16] at both the individual and community levels [17]. This review presents and discusses a new perspective of current approaches for treatment of schistosomiasis. Combination treatments with PZQ and other anthelminthic drugs as well as administration of antioxidant agents alone or as adjuvant in treatment of schistosomiasis are addressed and reviewed. 

## 2. Praziquantel: Mainstay Chemotherapy against Schistosomiasis

**Figure 1:**
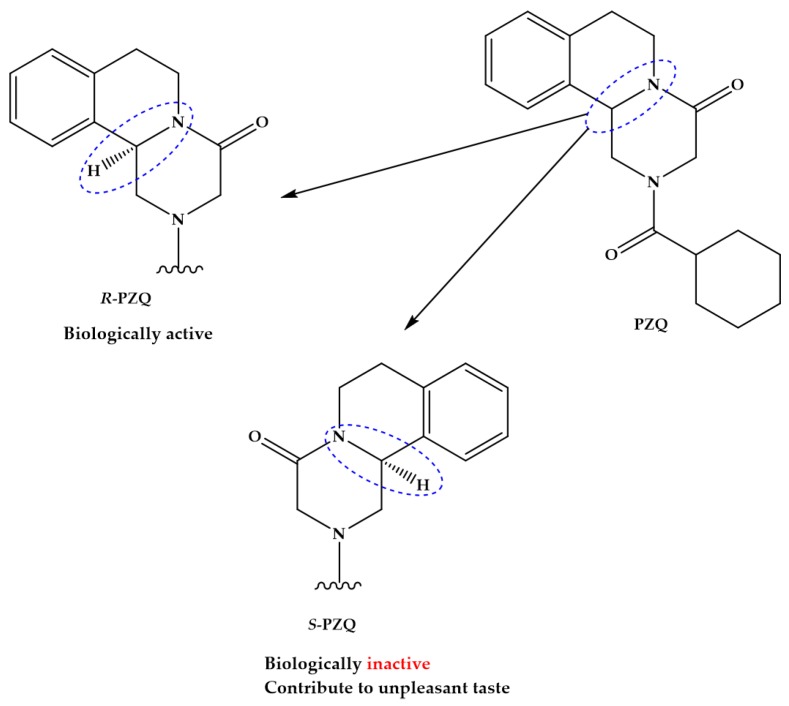
Chemical structures and characteristics of praziquantel and its enantiomers, *R*-PZQ and *S*-PZQ.

The pyrazino-isoquinolone compound praziquantel (PZQ, Figure 1) is widely accepted and used for the treatment of all forms of schistosomiasis and, indeed, infections with most flatworm parasites [18,19]. PZQ is effective against all schistosome species and generally causes only mild and transient side effects [17]. Since 2006, many millions of doses of PZQ have been consumed, mostly in Sub-Saharan Africa; and it has been estimated that by 2018 as many as 235 million people will have been treated with PZQ [20]. Nonetheless, PZQ presents some drawbacks such as is distributed as racemate that includes equivalent proportions of biologically active *R*-PZQ and inactive *S*-PZQ enantiomers (Figure 1), consequently half of PZQ dose is pharmacology inactive; low solubility and passes through extensive metabolism via hydroxylation of the absorbed drugs to inactive metabolites [21,22]. The presence of *S*-PZQ, the inactive diastereosisomer, both contribute to the large size of the tablet and its bitter taste, which impedes compliance by patients, especially children [21,23,24]. Another major drawback of PZQ is its inefficacy against juvenile stages relative to mature worms, thus complete cure is unusual with a single dose of PZQ [22,25,26].

In spite of the comprehensive use of PZQ, the model of action against schistosomes and targets molecules of PZQ in schistosomes remain to be elucidated. Substantial evidence implicates calcium channels as targets because schistosomes display marked fluxes in calcium ion (Ca^2+^) following exposure to PZQ [27], that leads to tetany of the musculature [28] accompanies by damage to the tegument [29]. The hypothesis that PZQ perturbs Ca^2+^ channels is supported by findings of studies that employed calcium-channels blockers and cytochalasin D [30]. More recently, was demonstrated that PZQ is a GPCR ligand, with *R*-PZQ acting as an agonist at human 5-HT_2B_ receptor [31].

Reliance for many decades on singular drug has obviously raised legitimate concerns about PZQ-resistance [32]. Whereas widespread drug resistance to PZQ has not arisen, field and experimental isolates that exhibit significantly reduce susceptibility have been described, which foreshadows for emergence of resistance against parasites [33,34,35]. 

Despite the efficacy of PZQ in reducing morbidity, the reliance of treatment and control on this drug and the concerning with emergence of resistance, prompt the search for additional therapeutics or other control strategies [35,36,37,38]. Continuous efforts have been made in order to circumvent reliance on a single medicine through synthesis of derivatives of PZQ and investigation of their antischistosomal activity [39,40]. Unfortunately, however, these derivatives have generally not achieved improved activity compared to that of the parental drug. In most cases, the promising in vitro activity of candidate drugs cannot be extrapolated to accept in vivo activity, likely related to pharmacokinetics and metabolic profiles, the key determinants of in vivo efficacy [32]. Several morbidity control strategies are often combined, but they need to be improved for comprehensive integrated control programs. In addition, given the reliance on PZQ, it is necessary optimize the useful life span of PZQ, through investigation of the potential for administration of combined PZQ therapies, avoiding the emergence and evolution of drug resistance. Over the years, it has been emphasized that this strategy would reveal synergistic effects and therefore might improve the treatment of helminth infections.

## 3. Treatment of Schistosomiasis: Anthelminthic Drugs Alone or Combined

Rational combination chemotherapy was developed for tuberculosis and other bacterial infections [41]. It is used for chemotherapy of cancer and acquired immune deficiency syndrome (AIDS) [42], and for malaria [43,44]. The main aims of this therapeutic strategy are to achieve additive/synergistic therapeutic effect and to minimize or delay the appearance of drug resistance [41,42,43]. When synergism/additive effect is exhibited, achieving similar or even enhanced efficacy at lower doses can be expected, along with reduction in side effects [42]. In the case of schistosomiasis, ideally the associated drugs would exhibit divergent mechanism of action to PZQ and/or target the immature schistosomes to enhance cure and egg reduction rates, as well as pathologies associated with infection. Herein, we summarize the evidence from experimental studies, in vitro and in vivo, as well as human clinical trials involving combination of different anthelmintic drugs against schistosomiasis. Initially, we start to introduce the drugs used against the disease and their combination in experimental studies (in vitro and in vivo) as well human clinical trials.

### 3.1. Oxamniquine

**Figure 2:**
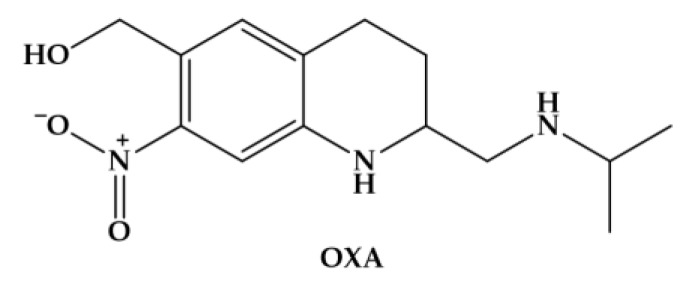
Chemical structure of oxamniquine (OXA), an antischistosomal drug used against *Schistosomiasis mansoni*.

Until recently, oxamniquine (OXA) (Figure 2) was the drug of choice for *Schistosomiasis mansoni* for many decades in Brazil [45]. 

The efficacy of OXA is confined to *S. mansoni*, unlike PZQ which is active against all schistosomes. However, like PZQ, OXA is more active against adult worms than the juvenile stages; and males are considerably more susceptible than females [46]. OXA induces less specific morphological alterations on parasite, and its hepatic shift occurs much more slowly post treatment. It has been considered that mode of action of OXA is related to inhibition of the nucleic acid metabolism. The hypothesis relies on activation of OXA by a single step, in which a schistosome enzyme converts the drug into ester that spontaneously dissociates, resulting in electrophilic reactants capable of alkylation of schistosome DNA [16]. This drug is also converted to a reactive sulfate ester and the activating enzyme is a sulfotransferase [47]. Resistance in *S. mansoni* to OXA is controlled by a single autosomal recessive gene. Anderson coworkers demonstrated, using two strains of *S. mansoni* from Brazil that differed 500-fold in sensitivity to OXA, and by Mendelian-type genetic crosses followed by linkage mapping, genomic sequencing and X-ray crystallography that a schistosome gene that encodes a sulfotransferase responsible for activating the prodrug to its active form, which in turn intercalates into the genome and interrupts nucleic acid synthesis [48]. 

As PZQ, OXA is safe and side effects are limited to mild but transient dizziness [49]. Although, low cure rates obtained for *S. mansoni*-infected patients treated with OXA have been described repeatedly, thus far, these do not constitute a significant concern for public health [15]. 

Combination therapy with OXA and PZQ have been used since 1980s, both in the laboratory and the clinic. However, findings with this combination are not clear, and would benefit with further investigation and stricter criteria [50]. In 1983, Shaw and Brammer investigated combinations of PZQ and OXA in adult *S. mansoni* in mice [51]. The findings were encouraging with respect to enhanced antischistosomal effects, more marked than either drug alone [51]. This combination regimen of low doses (1/3 curative doses of both drugs) was more effective four hours post-infection (p.i.), while administration at 5 weeks p.i. resulted in more marked worm burden reduction than PZQ and OXA monotherapy [52]. These results might be related to an early effect on the developing gonads of the schistosomula when mice were treated with OXA 4 h p.i. and interference with copulation when PZQ was given 5 weeks p.i. [52]. In a different study, low dose combinations of OXA-PZQ were active on different strains of *S. mansoni*—Venezuelan YT and SM and Brazilian BH, Complete elimination of all three strains was accomplished. When given alone, high doses of OXA or PZQ were required to obtain similar efficacy obtained with combined low-dose formulations. From these reports, it is reasonable to conclude that a combination of OXA-PZQ acts through synergism [53]. 

Human clinical trials were undertaken to evaluate efficacy of this combination during combination treatment in 158 schoolchildren aged 6–20 infected with *S. mansoni* in Malawi [54]. Findings from 102 children with *S. mansoni* infection and 56 children with *S. haematobium* infection were reported. Significant egg reduction (93–99%) was observed in *S. mansoni*-infected children treated with PZQ (15 to 20 mg/kg) and OXA (7.5 to 10 mg/kg). Combined treatment also markedly reduced numbers of eggs of *S. haematobium* passed in urine (97–99.2%), somewhat unexpectedly since OXA is generally considered not active against this schistosome species [54]. Some authors considered that several aspects of this trial may have influenced these unusual findings including (1) small number of individuals in the treatment groups; (2) co-infection with both parasites of half the participants; (3) the use of only egg counts as the only end point; (4) high infection intensities in the population, and (5) the collection of only one stool or urine sample specimen at one month post-treatment [15].

A similar clinical trial was conducted in children in Zimbabwe, which included 58 school age participants who were infected with *S. mansoni* and *S. haematobium*. PZQ (20 mg/kg) and OXA (10 mg/kg) achieved a cure rate of 89% for the *S. mansoni* infections [55]. Here the PZQ-OXA combination failed to cure *S. haematobium* infections with high egg count reduction found for both parasite species being attributed to PZQ in cases of *S. haematobium* infections [55]. The study design exhibited limitations (small sample size, among others) similar to those carried out in Malawi, although the study end points included both cure and egg reduction rates. In addition, multiple samples of feces and urine was examined in order to assess therapeutic efficacy [54], and accordingly the findings may be more solid than those from the clinical trial in Malawi.

### 3.2. Antimalarials

**Figure 3:**
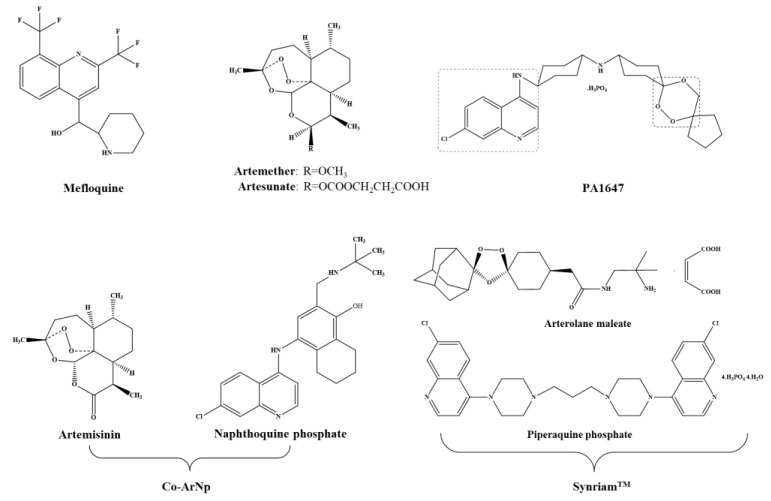
Antimalarials studied alone or in combined regimen on experimental infections and clinical trials. PA1647 and Synriam^TM^ are trioxaquines and trioxalanes respectively. Note that PA1647 contain two antimalarial pharmacophores: a 4-aminoquinolone and a 1,2,4-trioxane.

Antimalarials have been tested against schistosomiasis either alone or combined. Figure 3 depicts the chemical struct of some of these compounds.

#### 3.2.1. Artemisinins Derivatives

In searching for alternatives to PZQ, studies have been undertaken with semi-synthetic derivatives of the sesquiterpene lactone, artemisinins, including artemether (ART) and artesunate (AS) (Figure 3) which possess activity against the human schistosomes [15]. Dissimilar to PZQ and OXA, ART and AS exhibits high levels of activity against juveniles while the invasive stages and adult worm are less susceptible. Moreover, unlike OXA, adult female worms are somewhat more susceptible to ART than male worms [55]. Although the exact mechanism of action of artemisinin against schistosome remains elusive, evidence suggests that ART alters glycogen content in schistosomes [56], accompanied by morphological alterations similar to those induced by PZQ [55]. Exposure of schistosomes in medium containing ART plus hemin kills the worms while exposure of compounds alone does not. Hence, ART might be activated by hemin which subsequently cleaves an endoperoxidase bridge to liberate free radicals which, in turn, form covalently bind to schistosome proteins [57]. Also, hemin enhances in vivo efficacy of ART against *S. mansoni* [58]. Artemisinins are not only safe [59], but also possess a discrete mode of action of PZQ [60]. In fact, in a recent meta-analysis it was confirmed that artemisinins used in combination with PZQ have the potential to increase the cure rates in schistosomiasis treatment [61].

##### Arthemeter

Efficacy of PZQ-ART combinations were assessed in performed in different host-parasite models [62,63]. The reports revealed consistently higher worm burden reductions following treatment with combined regimen compared to PZQ or ART alone. In rabbits infected with juvenile and adult *S. japonicum*, treatment with 50 mg/kg of PZQ and 10 mg/kg of ART with one day apart significantly reduced worm burden (82%) compared with PZQ and ART monotherapies (66% and 44%, respectively) [62]. Similar results were seen with rabbits infected only with adult *S. japonicum* [63]. Hamsters infected with juvenile and adult *S. mansoni* were simultaneously treated with 75 mg/kg of PZQ and 150 mg/kg of ART. Administration of the combined regimen reduced worm burden by 77% which was significantly higher than 2% reduction achieved with PZQ monotherapy, but, it was not significantly different from 66% reduction obtain with ART alone [64]. Mahmoud and Botros [64] investigated the therapeutic effect of PZQ-ART combination in mice infected with *S. mansoni* in differential developmental stages. The antischistosomal effect of a single dose of ART was similar for adult and juvenile *S. mansoni*. Histopathological changes were evaluated. In contrast to results observed in hamsters, administration of PZQ-ART combination led to >90% worm reduction which was not statistically significant when compared with 95% achieved with PZQ monotherapy. Nonetheless, the impact of combined treatment regimen on *S. mansoni* eggs was impressive, with complete absence of eggs from tissues with minor histopathological changes in the liver. Although the few residual worms recovered from groups receiving the PZQ-ART combination were almost sterile and incapable of oviposition, this evidence alone might not explain the complete absence of eggs and granulomas from tissues [64]. Free radical liberation plays a role in drug induced-immune responses [64].

The safety and efficacy of ART-PZQ in different regimens for treating schistosomiasis japonica was assessed on a randomized double-blind, placebo controlled clinical trial in 196 Chinese patients for a 45-day period [65]. Administration of PZQ either or without ART during acute schistosomiasis was highly efficacious. Two end-points were included in trial, infection status (determined by stool examination) and blood chemistry. The combination of PZQ-ART in two distinct dosages (60 mg/kg and 120 mg/kg of PZQ plus 6 mg/kg ART) achieved parasitological cure rates of 98.0% and 97.7% (*p* > 0.05), respectively. Nonetheless, these results were not statistically different from those obtained in control group (PZQ/placebo, 96.4% and 95.7%, *p* > 0.05) [65]. Apparently, in contrast to laboratory findings with rodents, the combination of ART-PZQ did not improve the efficacy in infected people, when compared to PZQ alone. 

##### Artesunate

Although AS caused a significant reduction from egg tissue in comparison to PZQ, curiously, AS did not markedly reduce numbers of female parasites. AS appeared to impair fecundity of the adult females rendering them sterile rather than limited release of eggs [66]. Administration AS-PZQ to mice infected with *S. mansoni* significantly reduced in total worm count with complete eradication of female worms and tissue egg count in comparison to monotherapy with PZQ or AS. The action of the AS-PZQ combination may relate to the effect of AS in adult females, leading to a reduction of eggs with efficacy of PZQ against adult worms that results in elimination of the worms. In addition, Abdin and co-authors investigated effects of AS-PZQ on schistosome thioredoxin glutathione reductase (TGR) and cytochrome c peroxidase (CcP). They suggested that AS activity might be mediated by expression of these genes; by contrast, PZQ failed to influence expression of these genes. The loss of these two defensive enzymes likely renders the parasite vulnerable during its different stages to reactive oxygen species (ROS) [66]. Although CcP, a mitochondrial enzyme expressed in adult schistosomes that protects against endogenous and exogenous H_2_O_2_, it is unlikely that CcP exerts a general effect on peroxidation outside the mitochondrion [66]. Both CcP and TGR might be developed as drug targets since TGR especially displays functional and biochemical differences between redox metabolism and the human host [66,67].

Efficacy of a AS-PZQ combination was evaluated in a non-blinded open-label trial in Senegal that enrolled 110 local residents who were stool-positive for *S. mansoni* infection, aged 1 to 60 years. These participants were assigned groups that received either a single oral dose of PZQ (40 mg/kg), the recommended dose regimen of AS (4 mg/kg followed by four daily doses of 2 mg/kg), or a combined treatment. Parasitological parameters including cure and egg reduction rates were evaluated at 5, 12 and 24 weeks following treatment using two Kato-Katz thick smears taken from the same, single stool specimen. Since Senegal is an area of intense transmission of *S. mansoni*, reinfections occur frequently and quickly. In this regard, the therapeutic efficacy during first 5 weeks will be discussed here. Despite treatment with AS-PZQ resulting in cure and reduced numbers of eggs higher (69% and 89%, respectively) than with monotherapy, the egg reduction rate was similar to PZQ alone (84%), however, it was higher than AS alone (59%) [68]. AS also failed to affect the number of eggs. 

A combination of AS-PZQ was evaluated in a double-blind, randomized, placebo-controlled study in Gabon that enrolled 296 children aged 5 to 13 infected with *S. haematobium*. By 8 weeks post-treatment, egg numbers in urine in two consecutive urine samples were ascertained. A cure rate of 81% (95% confidence interval, CI) was observed in the group treated with combined AS-PZQ which is not significant when compared to the cure rate for PZQ monotherapy (73%, 95% CI). In addition, the cure rate of 27% (95% CI) obtained in the AS monotherapy group was also not significantly different to the placebo (20%, 95% CI), which was attributed to day-a-day variation in numbers of eggs in the urine [69].

Administration of AS-PZQ was assessed in the treatment of urogenital schistosomiasis (UGS) in several villages of Nigeria [70,71]. Inyang-Etoh and colleagues enrolled 312 randomly selected schoolchildren aged 4 to 20 years. Groups were treated with PZQ-placebo, AS-placebo, PZQ (40 mg/kg), AS (4 mg/kg), or a combination of PZQ (40 mg/kg) and AS (4 mg/kg) [70]. Cure and egg rate were assessed by examination of urine for schistosome eggs. As observed in Gabon [69], high cure and mean ova reduction (88.6% and 93.6%) rates were obtained with the combination of drugs while PZQ achieve a cure rate 72.7% and AS 70.5%. However, significant differences were not apparent among cure rate with AS-PZQ, and PZQ with or without placebo. These results reinforce that PZQ is maximally active again adult schistosomes. The differences between cure rates with PZQ-AS compared to the AS-placebo support the notion that AS has fewer schistosomicidal activity against adult schistosomes [70]. Similar findings were seen in a nearby village among 70 children diagnosed with UGS following administration of the AS-PZQ combination, where treatment with a combination of AS (4 mg/kg/day over 3 days) plus PZQ (40 mg/kg once) and single oral dose of PZQ (40 mg/kg). Number of eggs in urine one month following drug administration were ascertained. The AS-PZQ combination lead to significantly higher cure (85.7%, 95% CI) compared to monotherapy with PZQ (51.4%, 95% CI). These results are consistent with those described above and might be attributed to a synergistic effect of these two drugs, but using a different mode of action [71]. Cure rates with the same combined therapy differed among villages, which may have reflected divergent susceptibility profiles of parasite genotypes across the endemic range of schistosomiasis.

##### Artesunate Combined with Sulfamethoxypyrazine/Pyrimethamine

The antischistosomal activity of a new combination therapy AS-sulfamethoxy-pyrazine/pyrimethamine (AS-SMP) was evaluated among 800 school-aged children infected with *S. haematobium* [72]. Children were allocated into groups and treated with PZQ alone and AS-SMP, and urine samples were examined on days −1, 0, 28 and 29. A higher cure rate was achieved with PZQ group (53%), suggesting that AS-SMP (43.9%, *p* = 0.011) is not effective at least against adult worms or even with this schistosome species. A moderate enhancement of egg reduction was seen in PZQ groups (95.6%) in comparison to AS-SMP (92.8%, *p* = 0.096) [72]. This might relate to the fact of the dose administered corresponded to doses used for malaria. Additionally, the study revealed that safety and tolerability profiles of this combination were similar to PZQ [72]. It will be necessary assess if increasing the dose would enhance the antischistosomal efficacy and retain safety and tolerability. Efficacy and safety of AS-SMP should be evaluated for the other schistosome species [72].

##### Artemisinin and Naphthoquine Phosphate

A novel oral antimalarial drug, naphthoquine consisting in a combination of naphthoquine phosphate and artemisinin (Co-ArNp) (Figure 3) exhibits activity against *S. mansoni* in vivo [73]. The nature of the specific pharmacodynamic interaction between artemisinin and naphthoquine phosphate in the formulation was not clear, however co-administration of both drugs induced significant synergistic interaction [73]. Oral administration of Co-ArNp in a single dose of 400 mg/kg in mice infected with *S. mansoni* (Egyptian strain) on day 7 p.i. reduced the worm burden by 95%. Increasing the oral dose up to 600 mg/kg on day 21 p.i. resulted in an elimination of all female worms before they commenced laying eggs. In addition, the combined regimen provided significant reductions in the hepatic and intestinal tissue egg loads, and induced significant alterations in oogram pattern. These alterations might be attributed to the activity of artemisinin, which might be augmented by co-administration with napthoquine phosphate harming the female worms and oviposition [74]. Despite these promising findings in mice, it was necessary to study the toxicity of this combination. Clinical trials will be required to determine whether artemisinin/PZQ combination therapy offers advantages and whether the inevitable higher cost of such a combined treatment makes it practicable, and artemisinins (alone or in combination with PZQ) to treatment of schistosomiasis is the risk of driving artemisinin resistance in malaria in areas where both diseases are endemic [75].

#### 3.2.2. Mefloquine 

Mefloquine (MFQ, Figure 3), an antimalarial agent, is considered one of the best antischistosomal drugs [76]. Similar to artemisinin, MFQ is also active against developmental stages of schistosomes. In adult worms, MFQ induced extensive, severe damage on tegument, musculature, digestive and reproductive systems [76,77,78]. It was anticipated that combinations of MFQ with other drugs would be more effective rather PZQ monotherapy. Therefore, evaluation of combine regimens of MFQ-PZQ and MFQ-artemisinin derivatives against schistosomiasis have been performed both experimentally (in vitro and in vivo) and in human clinical trials [79,80,81,82,83,84]. 

The efficacy of MFQ administered orally at single, multiple doses, or in combination with AS, ART, or PZQ was assessed in the *S. japonicum*-mouse model at 4 weeks post-treatment. Administration of MFQ (50 mg/kg or 100 mg/kg) in combination with ART or AS (100 mg/kg) totally eliminated female worms, especially in mice treated with combination of MFQ-ART. The better results were achieved using higher doses of 100 mg/kg. Elimination of females might be a valuable target since eggs are central of the pathogenesis of schistosomiasis [79]. Also, combined regimens achieved a significant decrease of total worm burden (76.7% for MFQ-AS, 1:1, 100 mg/kg and MFQ-ART, 1:1, 100 mg/kg: 87.8%) compared to monotherapies (AS 100 mg/kg: 59.8% versus MFQ 100 mg/kg: 67.9% and ART 100 mg/kg: 55.6%). This outcome suggests a synergistic effect between MFQ and artemisinin derivatives [79]. MFQ-PZQ was also evaluated in *S. mansoni* infected mice at the same doses as above and also at 400 mg/kg each. Higher total and female worm burden reductions (86.0% and 93.0%, respectively) were achieved only when either MFQ-PZQ at the highest dose were given simultaneously or MFQ was given 24 h prior to PZQ. At the lower doses of 50 and 100 mg/kg, combinations involving PZQ followed a day later with MFQ induced only moderate total worm burden reductions, 47.8–54.7%. The most impressive outcome was seen when PZQ treatment were followed by MFQ, which suggests that PZQ might play a role in the antagonistic effects when it was administered before MFQ [80]. In mice simultaneously infected with *S. mansoni* 14-day-old schistosomula and 49-day-old adult worms, administration of a daily dose of 100 mg/kg of PZQ and MFQ for two consecutive days markedly reduced worm burdens of immature and adult schistosomes (both >95%) as well as immature eggs in comparison to monotherapies (PZQ: 29.2% and 49.6%; MFQ: 41.9% and 67.4%, respectively). Additionally, histopathological examination on liver sections revealed that the combined regimen significant reduced granuloma diameter in comparison to monotherapies. Enhanced worm burden reduction might be correlated with healing of hepatic granulomatous lesion, since eradication of females led to inhibition of oviposition [81]. Abdlel-Fattah and colleagues obtained contradictory results from those described above. In *S. mansoni* infected mice treated 3 or 6 weeks with curative (400 mg/kg MFQ and 500 mg/kg PZQ) or subcurative (200 mg/kg MFQ and 250 mg/kg PZQ) doses, MFQ monotherapy was more potent than the combined regimen in full dose regimen and PZQ monotherapy in its low regimen [82]. The authors attributed the additive/synergistic effect of PZQ and MFQ, observed from other studies [80,81] to the simultaneous presence of juvenile and adult worms, a difference that might explain the failure to achieve an additive effect in other studies [82]. 

MFQ-PZQ combinations were evaluated in clinical trials. A randomized, exploratory open-label trial was carried out in Cote d’Ivoire where 83 schoolchildren infected with *S. haematobium* were divided in four groups and treated with: (i) MFQ (single dose of 25 mg/kg); (ii) AS (4 mg/kg daily for 3 days); (iii) MFQ-AS (single dose of 40 mg/kg) and iv) PZQ (single dose 40 mg/kg). PZQ achieved highest cure rate (83%) followed by combine regimen (61%) while monotherapies of MFQ and AS only resulted in lower cure rate (21% and 25%). In children, concurrently infected with *S. haematobium* and *S. mansoni*, the treatment with PZQ and MFQ-AS resulted in high cure rates of 83% and 75% and egg reduction rates, 97% and 96% respectively. Despite the higher cure rate with PZQ monotherapy, the combination of MFQ-AS, administrated in accordance with currently recommended malaria treatment, showed encouraging results in co-infected children [83]. Keiser et al. assessed the efficacy and tolerability of similar treatment described, with the inclusion of MFQ-AS (3 × (100 mg/kg AS + 250 mg/kg MFQ)) combined with PZQ (MFQ-AS-PZQ) [84]. Urine from 61 children was collected before, and on days 21–22 and 78–79 after first dosage. Unexpectedly, on both follow up a marked reduction in the intensity of infection with high egg reduction rates but low cure rates were recorded in the three treatment groups [84]. The investigators suggested that the lower cure rates obtained with PZQ might reflect that children treated with this drug had high infection intensities before the drug administration. It was expected that combination regimens achieved higher cure rates since drugs have antischistosomal activity and, moreover, act on different developmental stages. These results might be explained by default of assessment of viability of excreted eggs since they did not count dead eggs; thus, cure rates might underestimate the true situation. Notably, the findings contrast with those from some other reports. From earlier reports with similar treatment schedules, it was expected that higher cure rates combining antimalarial (AS, ART and MFQ) with PZQ would be obtained compared to PZQ alone [73,74,81]. The authors observed on first follow up, a conclusion whether the addition of MFQ and/or AS would expand the activity profile of PZQ targeting juvenile schistosomes could not be made [84]. Overall, PZQ monotherapy was the best tolerated treatment, which might be explained by the only one type of drug administered. In groups treated with combination regimens, more than 90% of patients reported side effects, but it is unclear whether the adverse effects related to PZQ or systemic exposure of the antimalarials [84]. 

The role of MFQ in combined regimens should be investigated further in order to elucidate effects of these kind of combinations on acute schistosomiasis, and to characterize potential adverse effects of combination regimen of PZQ and MFQ.

#### 3.2.3. Trioxalanes/Trioxaquines

##### Synriam™

The piperaquine phosphate was recently developed as an antimalarial drug and manufactured by Ranbaxy (Goregaon, India) as Synriam™ (SYN, Figure 3) [85]. Administration of SYN to mice infected with *S. mansoni* significantly reduced worm burdens similarly to piperaquine phosphate (up to 80%) while arterolane was less effective (31% reduction in worm burden). In addition, SYN, unlike PZQ exhibited antischistosomal activity against schistosomula and juvenile stages, indicating a potential for use in prophylaxis. Moreover, adult females are more susceptible to SYN than males that might be related to higher levels of haem inside its gut since SYN, like artemisinins, seem to be activated by this component. Moreover, administration of SYN in vivo led to general improvement in liver pathology and smaller-sized granulomas that might be a consequence of failure of eggs to produce key antigen(s) to stimulate dendritic cells/T cells [86]. It will be informative to assess the efficacy of SYN against the other human schistosomes.

##### PA1647

Trioxaquines (TXO) were developed against malaria and was also reported a dual mode of action: alkylation of heme by the trioxane entity and stacking of heme through aminoquinoline moiety leading to the inhibition of hemozoin in vitro [87,88]. Mice infected with *S. mansoni* were treated in day 21 p.i. with a combination PZQ and trioxaquine PA 1647 (Figure 3). In treatment with four oral doses of 25 mg/kg, the reduction of schistosomula burden was 73% while in PZQ and PA1647 monotherapy were only 24% or 18%, respectively. The results suggested an additive or synergistic effect against schistosomula. Due to this promising effect of PZQ and PA1647 against schistosomula, combinations of these drugs should be considered for clinical trials and might be relevant for use as chemoprophylaxis against schistosomiasis [89].

### 3.3. Other Combinations 

**Figure 4:**
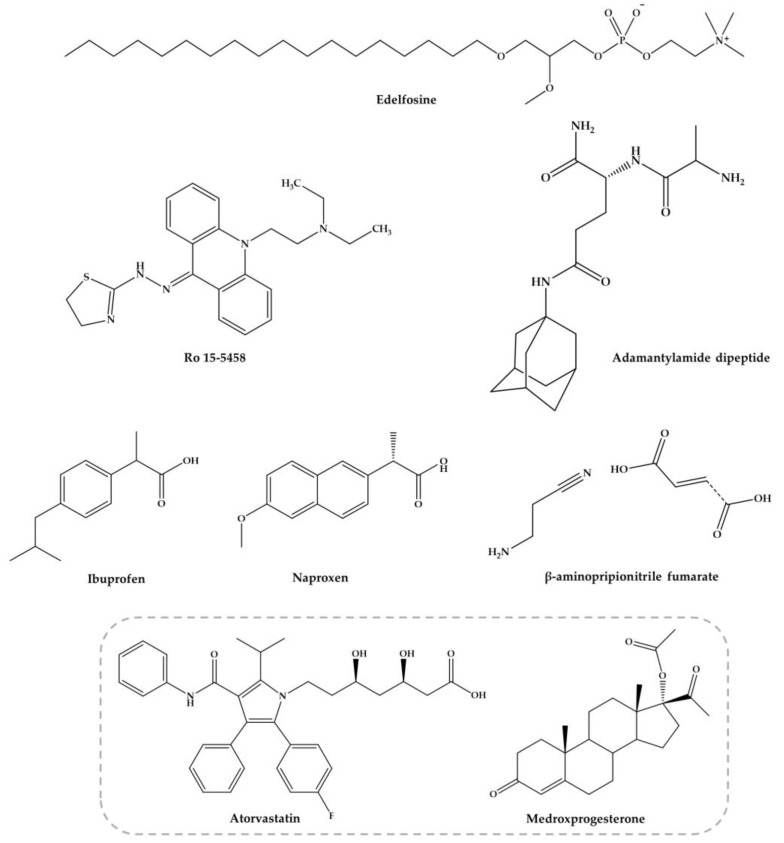
Several compounds within different properties against experimental *S. mansoni* infection. Compounds in the grey square were studied in combination, and others were studied alone and combined with PZQ.

The other combinations with nucleosides, acridine derivatives, anti-inflammatory agents, edelfosine, dipeptides, antifibrotic agents among others (Figure 4), are discussed below. 

#### 3.3.1. Nucleosides

In late 1980’s, El-Kouni and colleagues [90,91,92,93] evaluated treatment of schistosomiasis by purine analogues but using the combination with nucleoside transport inhibitors. This alternative strategy emerges since unlike humans, schistosome lack de novo purine biosynthesis (required for synthesis of DNA and RNA) and dependent on the salvage pathways for purine [94]. By blocking or interfering with the parasite purine salvage pathway using purine analogues, schistosomes can be selectively deprived of vital purines. Only a few analogues were tested since they are either not efficiently metabolized to nucleotide level by parasite or are also toxic to mammals. Mice infected with *S. mansoni* were injected with combined regimen of nitrobenzylthioinosine 5′-monophosphate (NBMPR-P, 25 mg/kg per day for 4 days) and high doses of tubercidin (5 mg/kg per day for 4 days). Notably, these doses were highly toxic to parasite but not to the mice. The treatment resulted in an impressive decrease in the number and copulation of worms, which consequently decreased the number of eggs in the liver and intestine. All eggs found were dead. Histopathological examination of livers showed lesions with dead worms and regeneration of normal tissue around old granulomas [89]. Similar results have been achieved with this combined therapy against *S. japonicum* [91]. This combination was also assessed on advanced stages of schistosomiasis by administration drug combination on 5, 6, 7 and 8 weeks p.i. in heavily infected mice. In the long-term progress of the disease (22 weeks p.i.), the efficacy of combination therapy was monitored. In this case, the reduction of number of worms and eggs in liver and small intestine were less evident [91]. Additionally, the combined therapy was effective in preventing the formation of novel egg granuloma although activity was not seen against existing granuloma. In another study, the therapeutic efficacy of NBMPR-P in combination in mentioned dose with potential antischistosomal purine analogues, nebularine (37 mg/kg/day), 9-deazaadenosine (1 mg/kg/day), toyocamycin (1.6, 1.8, 2.0 and 2.2 mg/kg/day for 4 days) and dilazep (25 mg/kg/day) was tested in *S. mansoni* infected mice. Administration of NBMPR-P combined with 9-deazaadenosine did not affect the parasites. However, combinations of NBMPR-P or dialazep with tubercidin or nebularine were highly toxic to schistosomes, achieving similar results as described above [93,94].

Despite these encouraging results in the laboratory, human trials with these drugs have not been reported, likely reflected potential toxicity risks associated to with these nucleosides.

#### 3.3.2. Acridine Derivatives

Ro 15-5458 (Figure 4) is an acridine derivative from class 9-acridanone-hydrazones that have been developed by Hoffman-La Roche (Basel, Switzerland) [95]. The possible synergistic and/or additive effect of PZQ with Ro 15-5458 was evaluated against two different strains of *S. mansoni* in mice, i.e., CD-susceptible and SO_4_-resistant strains. The treatment with a single curative dose of PZQ or Ro 15-5458 were also compared to those achieved by drugs in combination at doses corresponding to one-third of the curative dose of PZQ and Ro 15-5458. Combination of PZQ and Ro 15-5458 demonstrated to be beneficial as regard the percentage of parasite reduction and hepatic worm shift (99.4% to 100%, respectively in the CD-susceptible mouse strains, compared to 84.1% and 34.8% in the SO_4_ resistant strains) [95]. Moreover, it was observed a decrease in the number and size of granulomata with disappearance of pathological changes in hepatocytes. The authors considered that Ro 15-5458 has excellent antischistosomal properties and should be considered candidate for drug discovery and development pipeline [95,96]. However, thus far, no further investigations in order to evaluate mutagenicity and carcinogenicity of this compound as basis for possible clinical trials with humans.

#### 3.3.3. Anti-Inflammatory Agents

The nonsteroidal anti-inflammatory drugs (NSAIDs) were found to suppress the inflammatory process delaying hypersensitivity reaction in schistosomal hepatic granulomas and fibrosis [97]. Ibuprofen and naproxen (Figure 4) alone did not reduce significantly the worm distribution, egg load or change the oogram pattern when compared with the infected control [98]. Nevertheless, when administered in combination ibuprofen and naproxen with PZQ caused a slightly increase of percentage of dead ova (96.1% and 97.3%, respectively) at 16 weeks p.i. with a marked reduction of mature ova while PZQ alone reduce the number of worms to 95.6% [98]. These evidences demonstrated that they do not possess any antischistosomal activity, although they play a role in amelioration of biochemical and histopathological consequences related to intensity of infection [98]. Administration of these NSAIDs alone (200 mg/kg for two weeks) significantly reduced the granuloma diameter while has no effect on their type nor in serum levels of hepatic enzymes and circulating antigen. Treatment with ibuprofen and naproxen combined with PZQ (2 × 500 mg/kg) improved the parameters mentioned resulting in marked reduction in the mean granuloma diameter and circulating antigen which was more pronounced with naproxen than ibuprofen. These evidences suggest a combined action of PZQ in elimination of parasite and anti-inflammatory properties of ibuprofen and naproxen. Authors considered that treatment with NSAIDs is not preferable without PZQ but may be used as adjuvant in treatment of pathologies associated to infection [98].

#### 3.3.4. Edelfosine

Yepes and co-workers [99] study antischistosomal activity in vitro of a synthetic lipid compound, edelfosine (EDLF) (Figure 4), against schistosomula and its combination with PZQ in vivo. It has been reported that EDLF display anti-inflammatory properties [96] and modulate cytokine production such as interferon-γ (INF-γ), interleukin-2 (IL-2) and interleukin-10 (IL-10) [100,101]. This modulation might be relevant since cytokine production by host blood cells after stimulation with parasite antigen reflects a dominant T helper 1 (Th1) response during acute phase, producing interferon-γ (IFN-γ) and IL-2. Following parasites maturate, mate and produce eggs was followed by a developing egg antigen-induced regulatory T cell and T helper 2 (Th2) response, that downregulates the production and effect functions of the pro-inflammatory Th1 mediators accompanied by granuloma formation [102,103].

In a preliminary experimentally studies EDLF induce interruption of oviposition in vitro as well as significant reduction in worm burden in vivo being most effective against male worms [104]. Contrary to PZQ, EDLF is active against schistosomula of *S. mansoni*. In addition, authors study the effects of the combination of PZQ (100 mg/kg/day) plus EDLF (45 mg/kg/day) administered in *S. mansoni* infected mice daily 3 days prior to infection until eight days p.i. Combine regimen not only acts on parasite through the elimination of developmental stages; as well on histopathological parameters inducing reduction of hepatomegaly, granuloma size and immunological effects downregulation of Th1, Th2 and Th17 responses reflecting in inhibition granuloma development and up and down-regulation of IL-10 on early and late post-infection times, respectively. Consequently, this regulation potentiates anti-inflammatory actions and favoring resistance to re-infection. In addition, reduction in the number of blood granulocytes in late post-infection in comparison to infected untreated animals [99]. Taken these together it has been suggested that this combine regimen treatment may provide a promising and effective strategy for a prophylactic treatment of schistosomiasis.

#### 3.3.5. Antifibrotic Agent, β-Aminopropionitrile

The combination with non-schistosomal drugs such as β-aminopropionitrile-monofumarate salt (Figure 4) with PZQ was evaluated by Egyptian researchers in mice infected with *S. mansoni* [105]. The findings reveal that the combined regimen reduced total worm burden reduction (100%) and markedly reduced the egg load in the liver and intestines. Moreover, combination regimen revealed the highest score of resistance to reinfection, compared to the other groups given each drug alone [105]. Similar results were achieved with combine regimen of PZQ and β-aminopropionitrile (BAPN) in *S. mansoni* infected mice [106]. Modulation of granuloma formation by combined antifibrotic/PZQ therapy significantly alters the process of egg granuloma formation and alleviates the host resistance to challenge infection. Treatment results in relatively small sizes granuloma in comparison to large and irregular form of granulomas detected in intestinal tissues on control mice. However, the mechanism by BAPN reduces the number of liver granulomas is still unclear. In addition, treated mice with combine regimen showed decreased liver and spleen weights and a significant reduction in the number of eggs trapped in both liver (86%) and the intestine (99.1%), in comparison to untreated mice and those given PZQ alone [106]. According to authors, these results suggested that administration of PZQ combine with BAPN might also be useful as adjuvant in amelioration of pathologies associated to infection. 

#### 3.3.6. Adamatylamide Dipeptide

Botros et al. [107] investigate the possible use of adamantylamide dipeptide (AdDP) (Figure 4) as adjuvant therapy to PZQ in mice infected with PZQ-insusceptible and susceptible *S. mansoni* isolate in a trial to increase the susceptibility of this isolate to the drug. Seven weeks p.i., the experimental group received AdDP (5 mg/kg) in addition to PZQ in reduced dose (3 × 100 mg/kg) and groups received PZQ and AdDP alone. In mice infected with PZQ-susceptible and insusceptible *S. mansoni* isolates, intraperitoneal injection of AdDP alone did not significantly reduce the total number of worms suggesting that dipeptide did not present antischistosomal activity. Treatment with AdDP and PZQ in reduced dose resulted in significantly higher antischistosomal efficacy than PZQ in reduced dose, demonstrating that AdDP reduced the effective dose of PZQ. This efficacy obtained together with granuloma diameter reduction and diminution in percentage of fibrotic areas was also comparable to that observed in mice treated with full dose of PZQ. The results might be related to synergistic effect of PZQ and AdDP when administered in combine regimes; in fact, AdDP enhance antischistosomal activity and ameliorate the hepatic inflammatory reactions [107].

#### 3.3.7. Atorvastatin and Medroxyprogesterone Acetate

Soliman and Ibrahim [108] conducted a study in order to evaluate the influence of long-term administration of lipid lowering agent atorvastatin (AV, Figure 4) combined with injectable contraceptive medroxyprogesterone acetate (MPA, Figure 4) on tegumental structure and survival of *S. haematobium* worms. MPA was administered intramuscularly (0.1 mg/kg) at days 7 and 35 p.i. followed by AV treatment regimen (0.9 mg/kg for 49 consecutive days) in *S. haematobium*-infected hamsters. Long-term administration of AV induced mild to severe morphological alterations, particularly in the tegument of schistosomes. Similarly, treatment with AV concurrently with MPA significantly increased tegumental damage and significantly reduce the total numbers of *S. haematobium* worms recovered from hamster infected (51.3%). No significant difference was found in both combine regimen and AV monotherapy (46.2%). Female worms were less susceptible to both drug regimens compared to males [108]. The investigators correlate significant reduction of the recovered worms as result of both treatments with tegumental damages induced, in addition to the possible influence of AV on biochemical pathway of the parasite [108]. Furthermore, tissue egg load and oogram pattern decreased in hamsters treated with the combination regimen which might be related not only to reduction of recovered worms but also with suppression of egg production due to inhibitory influence of AV on the enzyme 3-hydroxy-3-methoxyglutaryl-coenzyme A (HMG-CoA) reductase, which is critical for regulation of egg production by the parasite. Inhibition of HMG-CoA should be investigated since it might be considered as a potential drug target [108]. Studying schistosomal and mouse liver HMG-CoA reductase activity was also observed elevated quantity in the liver but very reduced in the parasite [109]. In other work, the parasite death by statins or specific RNAi of HMG-CoA is associated with activation of apoptotic caspase activity [110].

## 4. Antioxidants: A New Chemotherapy against Schistosomiasis?

**Figure 5:**
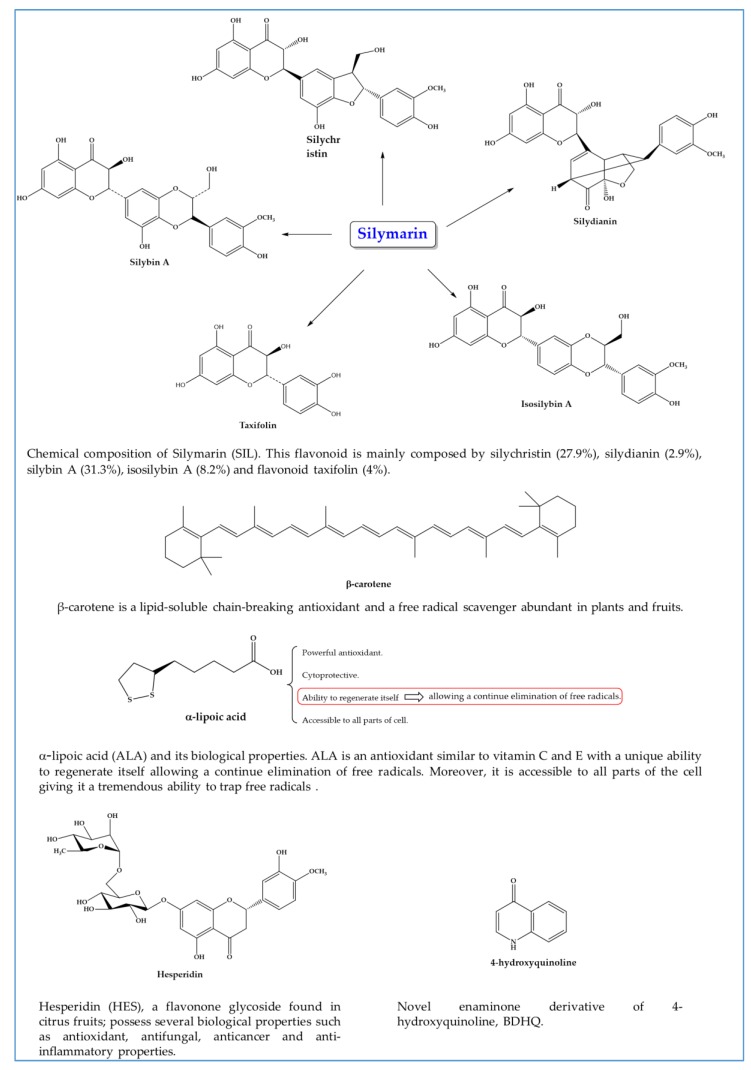
Chemical structures of compounds with antioxidant properties against schistosomiasis.

In recent times, studies evaluating efficacy of antioxidants, alone or concurrently with antischistosomal drugs targeting not only parasite but also pathologies associated to infection have been reported [111,112,113,114,115,116,117,118,119,120,121,122,123,124,125,126,127,128,129,130,131,132,133,134,135,136,137,138,139,140] (Table 1 and Figure 5).

Most antioxidants assessed have shown potential schistosomicidal activity both in vitro and in vivo, against mature [111,113,116,124,127,129,130,132,134,135] as well as immature [112,118,130,131] *S. mansoni* and *S. japonicum* [120] developmental stages, counteracting one of major drawback of PZQ. Even though the exact mechanism of action remains unclear, some antioxidants including pholoroglucinol derivatives and extracts of *B. trimera* affect motor activity of the worms in vitro. This phenotype is an insightful indicator of schistosomicidal activity, since it reveals perturbation/dysfunction of elements of the neuromuscular system [117]. In addition to movement, schistosomes use their neuromuscular systems to control the muscles of the oral and ventral suckers, which allow the worm attach to the host, musculature supporting internal organs including the reproductive excretory and digest tract, and maintenance of the female within the gynecophoral canal of the male [141,142].

**Figure 6:**
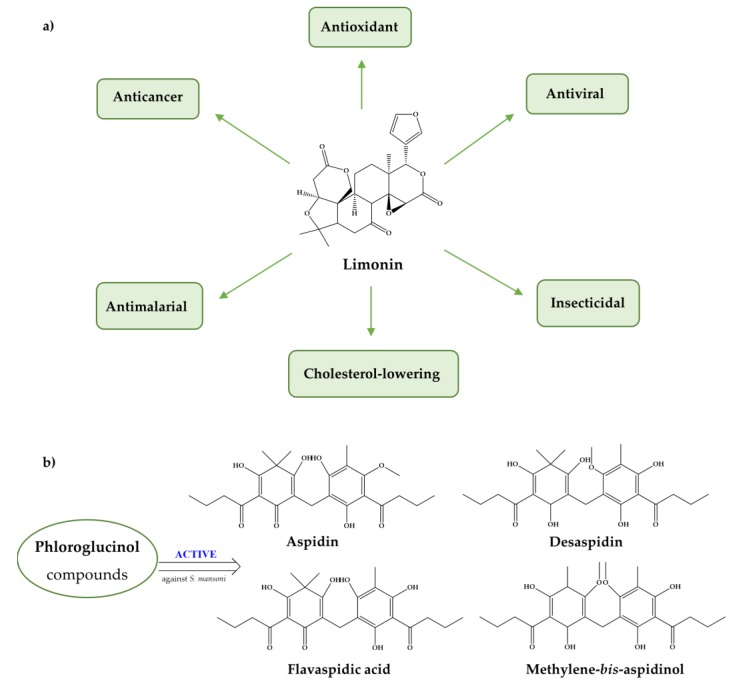
(**a**) Phytochemical limonin is abundant in citrus fruit and displays several pharmacological activities; (**b**) Phloroglucinol compounds, aspidin (APD), flavaspidin acid (FPA), methylene-*bis*-aspidinol (MbA), desaspidin (DSP), desaspidinol (DSPL) with activity against adult *S. mansoni* worms, they impede motor activity of the parasite [112].

Additionally, antioxidants such as limonin, phloroglucinol derivatives (Figure 6), and extracts of *B. trimera* and *A. sativum* induce severe tegumental alterations [112,113,117,127] which is noteworthy since the tegument plays a crucial role in host-parasite interactions, nutrient uptake for parasite growth and development, and protection against host responses [143]. Furthermore, several antioxidants have impaired worm coupling [112,130,132,137], a fundamental process for schistosome viability inside the host human and for establishing the infection. During pairing, the female is maintained in the gynecophoric canal in the male body for sexual maturation and egg production to occur.

The induction of separation of males and females by antioxidants reduce or even cease the release or production of the eggs [111,117,120,123,127,128,129], which are the major cause for the formation of inflammatory granuloma on target organs, and transmission of schistosomiasis [144]. In fact, the pathology associated with schistosomiasis is largely attributed to the intense of granulomatous inflammation and subsequent fibrosis induced by parasite eggs trapped in host organs such as liver, intestine and bladder. The toxic products released from egg destroys the host tissue cells and the antigenic material stimulates the development of larger inflammatory reactions leading to formation of granulomas around eggs [145]. As presumed, in the presence of parasite, the host immune system reacts in a manner that involves reactive oxygen species (ROS) leading to increase of oxidative process during the course of infection [145]. For example, eosinophils, one of the components of *Schistosoma*-induced hepatic granulomas, generate hydroxyl radical (OH) and the super oxide anion (O_2_) [146,147]. Several host organs, especially the targets, are affected by increased eosinophil peroxidase activity and imbalance in the antioxidant defense mechanism causing these organs to be shifted to a pro-oxidant state [145]. The ultimate aim of ROS generation may be killing the parasite eggs; yet, they alter liver homeostasis decreasing antioxidant defenses and increasing the liver enzymes such alanine aminotransferase (ALT), aspartate aminotransferase (AST) and gamma-glutamyl transferase (GGT) that are measures of liver affection [145]. 

**Figure 7:**
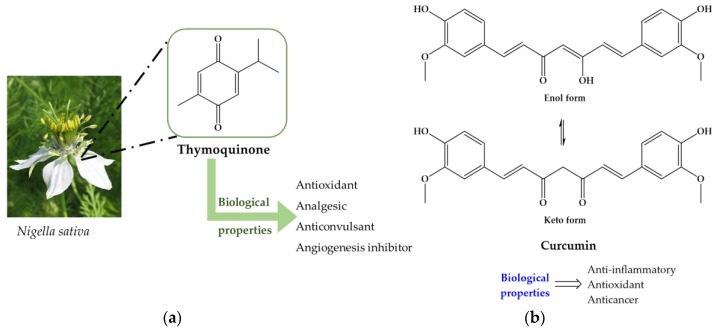
(**a**) *Nigella sativa* and its therapeutically active constituent, thymoquinone, responsible for diverse biological properties; (**b**) Chemical structures of curcumin and its biological properties. Curcumin is the principal curcuminoid of *Curcuma longa*. It is a diarylheptanoid, which is a natural phenol that exists in enolic form in organic solvents and as a keto form in water.

Mammalian cells has adopted a chain of antioxidant system, either enzymatic or not, to limit the overcome the harmful imposed by ROS. Enzymes such as superoxide dismutase (SOD), catalase (CAT) and glutathione peroxidase (GPx) are key players in defense against ROS. The SOD hastens the speed of dismutation of superoxide to hydrogen peroxide (H_2_O_2_). Afterwards, comes the action of catalase which transform H_2_O_2_ into H_2_O and O_2_ and tackle the chains of unsaturated fatty acids present in membranes and other macromolecules such as proteins. In cell, reduced glutathione (GSH) play a role in many biological processes, including the synthesis of proteins, maintenance of cellular activity, xenobiotics and reactive aldehydes detoxification (such as malonaldehyde, MDA), metabolism and cell acting protections against free radicals [145]. Some studies have suggested that a direct link to parasite load to intensity of inflammatory reaction and antioxidant activity, i.e., higher parasite load leads to intense immune response and decrease of antioxidant activity [146,148]. In this regard, there is a tendency to decrease the levels or even deplete GSH turning liver more vulnerable for adverse effects of ROS and other parasitic metabolites in the course of infection [148]. It has been described that patients treated with anthelmintic leading to eradication of worms are still unable to reverse hepatic fibrosis. Since morbidity associated to schistosomiasis are mainly resulted of liver fibrosis most interest has been focus on compounds that are capable to stimulate not only synthesis of antioxidant enzymes as well as enzymes associated to liver function. Administration of antioxidants on experimentally *S. mansoni* infected mice revealed that they are capable to restore activity of antioxidant and liver enzymes nearly to levels detected on controls [111,115,116,120,121,125,128,135]. Generally, increasing of antioxidant enzymes activity is accompanied by reduction on granulomas size and number which consequently improve liver architecture and functions [111,119,123,127,129,137,140]. The protective effects of antioxidants in some subcellular compartments may be due to its indirect antioxidant actions, e.g. stimulation of enzymes that promote the synthesis of other antioxidants or metabolize reactive species to non-radical products. On the other hand, parasites have developed antioxidant enzyme system, similar to humans, to defend themselves against ROS generated in immune host attack. In *S. mansoni* SOD, GR, GPX, CAT, glutathione-s-transferase (GST), thioredoxin reductase (TrxR) are major antioxidant enzymes that are involved in detoxification processes [149]. Therefore, the antioxidant defense mechanism of adult worms may represent potentially good target chemotherapy. It has been demonstrated that antioxidants, such as those present in extracts of *N. sativa* and curcumin (Figure 7), are able to inhibit parasitic antioxidant enzymes as well enzymes related to glucose metabolism (hexokinase, HK and glucose-6-phosphate, G-6-PDH) culminating in increase of oxidative stress that could turn render the parasite vulnerable to damage by host immune attack [131,137].

**Figure 8:**
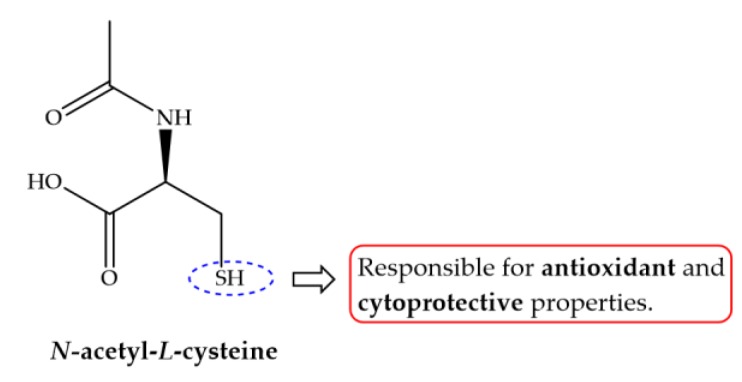
*N*-acetyl-l-cysteine (NAC) displays antioxidant and cytoproctive properties that are related to its free sulfhydryl group (blue elipse) which directly react with electrophiles including reactive radicals.

Besides antischistosomal activity and ability to restore liver functions, antioxidants modulate and immunomodulatory response promoting alteration in some cytokines [122,126,133,136,139]. Cytokines play an important role in immunomodulation during schistosomiasis. There are several events that are determined by a balance between different immune responses modulate by certain cytokines which are directed both against larval and adult stages of the parasite, as well as parasite eggs trapped in the tissues [150]. Eggs trapped in tissue secrete release antigens which are taken up by macrophages that stimulate T helper cells to secrete tumor necrosis factor α (TNF-α), which in turn drive to cell-mediate response attracting more immune cells around ova. As the granuloma becomes more organized, the T helper cells, produce different interleukins (IL) completing granuloma maturation towards the late stage of granuloma formation [150]. In case of murine schistosomiasis, TNF-α, IL-1, IL-2 and IL-12 are the causative IL in granuloma formation. In addition, granuloma cells comprise of macrophages, lymphocytes, eosinophils and release of profibrotic lymphokines such as IL4 [151] that stimulates fibroblasts to secrete collagen and other matrix proteins [152]. In experimentally *S. mansoni* infected mice treated with antioxidants shown controversial results, while treatment with curcumin resulted in low serum level of both IL-12 and TNF-α, *N. sativa* combined with ART or PZQ and *Allium sativum* showed significant increase on IL-2, IL-12 and TNF-α [127,133,136]. The dissimilarity of results obtained might be related to sampling time of experiment performed by Sheir and colleagues, which occurs after granuloma formation [131]. Moreover, treatment with *N*-acetylcysteine (NAC, Figure 8) increase synthesis of IL-10 which regulates the synthesis of several pro-inflammatory cytokines and is considered an efficient inhibitor of INF-γ, IL-12 and IL-4 [139]. 

Therefore, these responses produced by antioxidants might explain their role in reduce size and number of granuloma in infected host. Also, immunoglobins IgG and IgM have been shown to have a pivotal role in humoral response to schistosomal infection being related to periportal fibrosis and portal hypertension in patients with advanced schistosomiasis mansoni [153,154]. Administration of antioxidants to *S. mansoni* infected mice reduce serum IgG and IgM which might be directly linked to reduction of granulomas [127].

**Figure 9:**
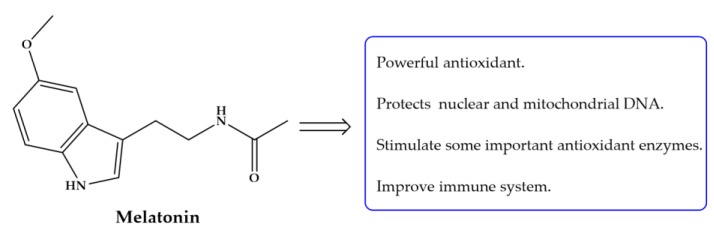
Melatonin (MEL) is a powerful antioxidant with a noteworthy protective action for nuclear and mitochondrial DNA due to efficacy as a radical scavenger. MEL may also stimulate activity of some key enzymes that participate in immunological functions.

Since parasitic antigens induce a host immune response, diverse *S. mansoni* antigens including adult worm antigen (SWAP), cercarial antigen (CAP) among other have been used to immunized experimental animals against *S. mansoni* either alone, in combination or with adjuvants. Unfortunately, most of studies have not achieved even a low significant protection against schistosome infection [155,156,157]. Immunization using SWAP and CAP alone or concurrently with melatonin (Figure 9) demonstrated that antioxidant enhanced SWAP efficacy which was confirmed by the absence of significant antibody (Ab) response in group immunized with SWAP + melatonin [120]. In different studies, it was also demonstrated that treatment with antioxidants augmented IgG response against SWAP, CAP and soluble egg antigen (SEA) [114,135]. These findings indicate the early and continuous antioxidant administration is responsible for the immunoprophylactic effect and may protect the liver against infection by reducing worm burden leading to improvement of liver function.

Generally, administration of antioxidants mixed or combined with antischistosomal drugs such as PZQ and ART, improved the parasitological and biochemical parameters described [115,123,125,132,133,138,139,140]. Considering that compounds present different mode of action it is reasonable hypothesized that those improvements are related with synergistic and/or cumulative effects of compounds when administrated in combination. 

Remarkably, antioxidants present not only antischistosomal activity but also induce restoration of organ target functions and improved host immunity, at least in animal model. Therefore, they should be considered as adjuvants in combine treatment of schistosomiasis. Nevertheless, many studies are required to fully understand the exact mechanism of antioxidants alone or combined against schistosomiasis. Additionally, clinical trials are required in order to assess if these results in animal model are reproduced in human host.

Interestingly, studies related to the effect of antioxidant against schistosomiasis haematobia are scarce. Despite there is no reliable animal model for this infection, it is extremely important conduct novel investigations of new therapeutic approaches against disease. *S. haematobium* is considered a biological carcinogen and in fact, the most adverse pathology associated to infection is bladder cancer [12]. Recently, it has been hypothesized about the role of parasitic reactive electrophilic compounds, e.g., estrogen-like metabolites, on initiation of squamous cell carcinoma (SCC) [10,158,159,160]. Possibly, these metabolites are capable to react with host DNA leading to formation of DNA-adducts and liberation of ROS, triggering a cascade of events that ultimately leads to development of SCC. Some evidences point out that antioxidants can prevent DNA damage [161] and block-cancer initiating process in case of breast cancer [162]. Therefore, it should be informative to assess their effect, alone or combine, in counteracting formation of these parasitic reactive metabolites.

## 5. Concluding Remarks

**Figure 10:**
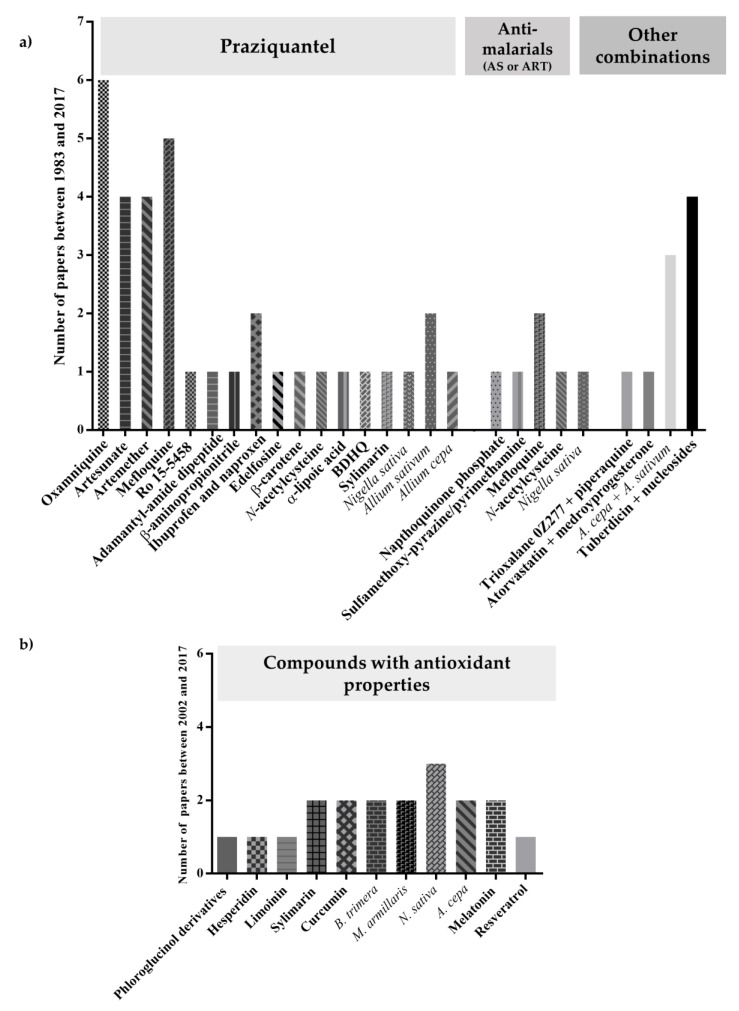
Graphical depiction of the number of publications dealing with (**a**) combinations between compounds with PZQ, antimalarials and other combinations; (**b**) compounds with antioxidants alone performed on both in vitro on in vivo cultured forms of schistosomes.

Nowadays, mass drug administration is main strategy for control of schistosomiasis but relies on the effectiveness of a single drug, PZQ. Although PZQ is highly effective, given by mouth and relatively inexpensive, PZQ has shortcomings that include lack of activity against immature schistosomes [1,17,19]. Moreover, PZQ alone does not lead to resolution of the histopathological damage characteristic of chronic schistosomiasis. Hence there is a need for new strategies, targeting not only parasite but also infection-associated pathogenesis. For the last years several combinations among different agents with PZQ and/or antimalarial and others are reported to represent encouraging leads for treatment approaches to overcome limitations of PZQ monotherapy (Figure 10a). Whereas PZQ combined with antioxidant agents (Figure 10) might or might not alter PZQ efficacy, combinations may nonetheless ameliorate tissue damage and infection-associated complications. Even though some antioxidants failed to inflict obvious harm to the schistosomes, they markedly reduced granulomatous inflammatory reactions as well as improved antioxidant and immunological responses to the infection. Alone or combined with other drugs, antioxidants might be valuable adjuvants to reduce morbidity and mortality of schistosomiasis. Moreover, natural antioxidants are considered safe for human use. Attempting new combinations of anthelmintic drugs with other biomolecules such as antioxidants provides new avenues for discovery of alternatives to PZQ.

## Figures and Tables

**Table 1:** Reports of antioxidant effects on developmental stages of schistosomes and infection in murine models.

Compound	Aim/Study	Treatment	Findings/Outcomes	Ref.
*M. armillaris*	Antischistosomal and antioxidant activity of essential oil on normal and infected *S. mansoni* mice in comparison to PZQ.	Oil 150 mg/kg orally from second week p.i., twice week for 6 weeks; PZQ 600 mg/kg, orally for 2 consecutive days, 8 weeks p.i.	Administration of PZQ and *M. armillaris* ameliorate the levels of GSH and lipid peroxides (MDA); Restored the activities of SOD and catalase; *M. armillaris* enhance antioxidant defense system reducing disease complications.	[111]
Limonin	Antischistosomal activity in vitro and in vivo harboring juvenile and adult *S. mansoni* worms.	Oral administration in a single dose of 50 or 100 mg/kg on day 21 p.i.; Same dose given on 56 p.i.	Limonin is more effective against immature stages rather adult worms also induce tegument alterations; Reduction of worm burden: at day 21 p.i. 70.0% and 83.3%; and day 56 p.i. 41.09 and 60.27%. Significant reduction in the hepatic (34.90% and 47.16%) and intestinal (46.67% and 56.1%) tissue egg load associated the oogram pattern with elevated dead egg levels; Also, ameliorate hepatic pathology with reduction in size and numbers of granulomas.	[112]
Pholoro-glucinol derivatives	Evaluation in vitro schistosomicidal activity of aspidin (AS), flavaspidic acid (FAA), methylene-bis-aspidinol (MbA) and desaspidin (DA) against *S. mansoni* adult worms.	AP-25 to 100 μM FAA-50 and 100 μM MbA-100 μM DA-25 to 100 μM	AP and FAA decrease motor activity with tegumental alterations while MBA and DA also decrease motor activity but without tegumental alterations. At highest concentrations viability of worms were similar to positive controls (PZQ); Egg production and the development of eggs produced were inhibited; Probably, in vitro activity is related to the inhibition of oxidative phosphorylation pathways.	[113]
Hesperidin	Evaluation of antischistosomal activity in vitro and in vivo and compared to PZQ. Effect on parasite antigens. Treatments were administered on 6th week p.i.	In vitro: 50, 100 and 200 μg/mL. In vivo: Hesp-600 mg/kg bw (6 injections, 2 injections per week for 3 consecutive week); PZQ (2 consecutive days with 500 mg/kg bw.	In vitro: At highest concentration, all males and females were dead while lower concentration had moderate effect. No activity on oogram pattern was seen. In vivo: Reduction of numbers of males, females and possibly worm pairs and total worm burden counts (47.5%) but was not higher than PZQ; significantly reduced tissue egg load. Augmented the mouse IgG response against soluble worm antigen protein, soluble egg antigen and cercarial preparation of *S. mansoni*.	[114]
α-Lipoic acid	Study combined effect of ALA with PZQ on liver fibrosis induced by *S. mansoni* infection in mice.	PZQ-500 mg/kg divided into 2 doses 9 weeks p.i.: PZQ (same described) + ALA (single dose 30 mg/kg) daily for two months.	Combine regimen results in reduction in the worm burden (ALA: 7.63 ± 1.49; PZQ: 6.13 ± 1.89; PZQ + ALA: 36.50 ± 10.80), egg count and granuloma size. Recovered the level serum of ALT, AST and GGT and increased the tissue level of GSH and decreased MDA (biomarkers of antioxidant function and stress oxidative, respectively).	[115]
Resveratrol	Investigate effect of Resv on oxidative stress imposed on liver, lung, kidney, brain and spleen of *S. mansoni*-infected mice.	20 mg/kg once daily for 2 weeks	Improvement of lipid metabolism and antioxidant profile by Resv which were not only restricted to liver but also other vital organs. Specific biomarkers of lung and brain homeostasis also showed remarkable improvement.	[116]
*B. trimera*	Assessment of antischistosomal activity agaisnt *S. mansoni* adult worms in vitro.	4 concentrations of 24, 48, 91 and 130 µg/mL.	Antischistosomal activity at highest concentrations with significant reductions in motility; Total inhibition in egg laying when parasites were exposure to sub-lethal concentrations and separations of all couples. Morphological changes on the tegument of worm’s males and females.	[117]
	In vitro and in vivo efficacy of aqueous fraction and dichloromethane extracts against schistosomula, juvenile and adult worms of *S. mansoni*.	In vitro: Same as above In vivo: Single doses 40 and 200 mg/kg of *B. trimera* and PZQ 4 after 3 and 30 days p.i. and 60 p.i.	In vitro: Similar results described. In vivo: *B. trimera* exhibits major schistosomicidal effects in vivo against immature and adult worms (significantly female worm, 68–75%, reduction and number of eggs/g in faeces); Significant reduction in relation to number and size of granulomas.	[118]
Melatonin	Assessment protection against oxidative stress induced by schistosomiasis mansoni.	3.55 mg/kg daily for 30 consecutive days starting from first day p.i.	Decreased in total leukocyte count: Markedly reduced the fibrotic areas, small diameter of granuloma with few collagen fiber depositions; ameliorate liver architecture and glycogen content.	[119]
	Establish an immunization program using *S. mansoni* adult worm antigen and cercarial antigen alone or concurrently with Mel in attempt to enhance efficacy against infection in mice.	30 μg/mL CAP or SWAP on first day and 20 μg/mL on 4th day p.i.; On 7th day all hamsters were infected. Mel same regimen as above.	Mel alone did not result decrease of worm burden reductions (CAP: 538%; CAP + Mel: 67.01%; SWAP: 56.4% and SWAP + Mel: 99.3%). Highly significant reductions in egg load in liver and alteration of oogram pattern: high percentage of immature eggs and few dead eggs. Improved the oxidative status in the immunized groups. No antibody response was observed in the groups immunized with SWAP + Mel while low antibody level was observed in CAP + Mel.	[120]
	Investigate oxidative processes in mice infected with *S. mansoni*	10 mg/kg, 2 weeks after *S. mansoni* infection until end of experiment; or daily for 30 days	Mel did not restore glutathione levels (although were tendencies for that); Increase SOD activity (but not statistically significant); Reduction of AST levels; Reduction of granuloma formation and highly protective against pathological changes not only in liver but kidney; Mel has multiple direct and indirect antioxidant actions and its ability to stimulate antioxidative enzymes and mitochondrial oxidative phosphorylation.	[121]
4-Hydroxy-quinolin-2(1H)-one (BDHQ)	Evaluation potential activity on murine schistosomiasis. For that mice were sacrificed on different weeks p.i.: 3 (for schistosomula) and 6 (for adult worms)	BDHQ: Lower dose—10 mg/mL for consecutive days; Higher dose on same regimen; PZQ: 2 times of 500 mg/kg 2 consecutive days on different weeks.	Antischistosomal activity against immature and mature worms; Destructive effects on the female and male genital systems; Antischistosomal activity may be due to its mixed cellular and humoral immunologic mechanisms, as demonstrated by the significant increase of serum levels of IgE and IFN-γ.	[122]
4-Hydroxy-quinolin-2(1H)-one (BDHQ)	Evaluation of antioxidant and antigenotoxic effects alone or combined with PZQ.	PZQ, 0 or 500 mg/kg BDHQ, 600 mg/kg PZQ (250 mg/kg) + BDHQ (300 mg/kg) for 2 consecutive days	BDHQ alone or combined resulted in highly significant reduction in total worm burden (7 weeks p.i. PZQ: 86.37%, BDHQ: 79.22%; PZQ + BDHQ: 91.84%; 9 weeks PZQ: 94.72%, BDHQ: 92.32%; PZQ + BDHQ: 95.54%), associated with significant reduction in the hepatic tissue egg load; Drugs alone reduced the granuloma size and inflammatory cells. These parameters were improved with combine regimen; Significant decrease in MDA level accompanied with highly increase in NOx level with combine regimen, in addition to increase in the activities of both SOD and CAT; Remarkable significant decrease in % DNA fragmentation reaching a level close to control; These suggest a synergistic action attributed to different mechanism of action of both drugs that achieved the same or higher levels of efficacy using smaller doses of either agent.	[123]
*Sylimarin*	Assessment of parasitological and biochemical parameters on *S. mansoni* infection in mice.	10, 20 or 25 doses of 10 mg/kg Syl suspended on carboxymethyl-cellulose at 55 days p.i.	Did not show antischistosomal activity; Reduced granulomatous and hepatic fibrosis. At acute schistosomiasis may result in a mild course of murine schistosomiasis and minimize the deleterious effects.	[124]
	Anti-inflammatory/antifibrotic effect alone and combined with PZQ.	Syl: —4th week p.i. (3 weeks before PZQ therapy) —12th week p.i. (5 weeks after PZQ); PZQ (7th week p.i.) Syl + PZQ	Syl alone: Partial decrease of worm burden (26.55 and 39.39%) and decrease hepatic tissue egg load with an increase in percentage of dead ova; Modulation of granuloma size and conservation of hepatic GSH. PZQ: Complete eradication of worm, egg and alleviated liver inflammation and fibrosis; Combine regime: Improvement of liver function and histopathology whether acute and chronic infection may due to a combine action of anti-inflammatory, anti-fibrotic actions, in addition to the antioxidant properties of silymarin. Syl did not interfere or affect the antischistosomal activity of PZQ. Worm burden reduction 97–100%.	[125]
*A. sativum*	Antischistosomal activity against *S. japonicum* cercariae in vitro and in vivo.	In vitro: 10^−2^ to 10^−6^ (*v*/*v*) concentration. In vivo: Pre-treated with garlic, then mice were infected.	Garlic oil displays marked activity agaisnts *S. japonicum* cercariae and may be used as agent to prevent *S. japonicum* (pre-exposure garlic oil at 10^−4^ and high showed total inhibition of infection).	[126]
*A. sativum*	Assess potency and the immunomodulatory response in enhancing the host immune system caused by *S. mansoni* in mice at different stages of worm.	100 mg/kg body weight from 1 to 7 days p.i., 14 to 21 or 1 to 42 days p.i.	Morphologic alterations in the parasite tegument; significant decrease in worm burden, hepatic and inestinal ova count. Decline in granuloma number and diameter; Reduction in serum TNF-α, ICAM-1, IgG and IgM after 7 and 42 days p.i.; garlic oil enhance host immune system.	[127]
	Ability of both oils to offser infectivity as well as metabolic disturbances induced by *S. mansoni* infection	5 mL/kg body daily separately for 8 weeks on healthy control and infected groups. On infected groups oil were given 24 h p.i.	Reduced worm burden (garlic: 67.56% and onion: 75.97%) and ova count; normalized liver functions enzymes; effect may be induced by improving the immunological host immune system and their antioxidant activities.	[128]
*A. sativum+ A. cepa*	Effect of both oils alone and mixed or concurrently with PZQ on biochemical parameters of experimentally infected *S. mansoni* mice.	*A. sativum* or *A. Cepa*, 2 g/100 g body weight daily for 45 consecutive days. PZQ: 500 mg/kg bw on 2 successive days 45 days p.i.	Significant reduction in worm burden (PZQ: 95.8%; onion: 66.29%; PZQ + onion: 99.1%; garlic: 73.41; garlic + PZQ: 99.3%; garlic + onion: 74.63; garlic + onion + PZQ: 99.7%); Reduction hepatic and intestinal eggs and oogram count; Suppression in granuloma tissue formation and diminutive histopathological changes; Improvement of liver architecture and attenuated the decrease of tissue antioxidant enzymes	[129]
	Antischistosomal activity in vitro against *S. mansoni* miracidia, cercariae, schistosomula and adult worm. Effect in vivo on lipid peroxidase and antioxidant enzymes.	In vitro: 0.5–5 ppm In vivo: Same described above.	Lethal effect of both antioxidant against all developmental stages; Inhibition of coupling; Powerful reducing capacity demonstrated in DPHH radical scavenging and NO; Both plants enhance host antioxidant system indicated by lowering in lipid peroxide and stimulation of SOD, CAT, GR, TrxR and SDH enzyme levels which could turn render parasite vulnerable.	[130]
	Antischistosomal activity against miracidia, cercariae and adult worms in vitro. Effect on some antioxidants enzymes.	In vitro: Serial concentrations (0.5–5 ppm) for miracidia and cercariae. Adult worms, 10–110 ppm.	Antischistosomal activity against miracida and cercariae; Separation of coupled worms; Inhibition of egg laying by adult female worms; Significant inhibition of parasitic antioxidant enzymes (SOD, GR and GPX) and enzymes glucose metabolism (HK and G-6-PDH), higher in males than females.	[131]
*Nigella sativa*	Effect in protection against oxidative stress in experimentally infected mice with *S. mansoni*.	*N. sativa* oil (1.14 g/kg orally) for 30 consecutive days from first day p.i.	No suppressive effect on granuloma formation in intestine; Did not improve the liver architecture; Noticeable degree of protection represented in less severe pathological changes, particularly the frequency of inflammatory reactions.	[126]
	Study effect the oil on liver functions and antioxidant ability on experimentally infected mice with *S. mansoni*.	2.5 and 5 mL/kg orally either alone or in combination with PZQ (500 mg/kg for 2 consecutive days)	*N. sativa* alone: Reduce the number of *S. mansoni* worms in the liver; Total worm burden: 22% e 32%, respectively, while PZQ: 98%; Decreased total number of ova deposited; Increased the number of dead ova; Reduced the granuloma markedly; Partially correct alterations in serum levels of ALT, GGT, activity as well as the Ab content. Failed in the liver restore either LPD and GSH content or LDH (lactate dehydrogenase) and SOD activity to normal level. *N. sativa* + PZQ: Improved most parameters with most prominent effect was further lowering in dead ova number over that produced by PZQ. Total worm burden: 98% and 99%.	[132]
*Nigella sativa*	Investigate immune mechanism possibly involved in the amelioration of histopathological changes in liver of *S. mansoni* infected mice treated alone or in combination with ART or PZQ.	*N. sativa*: orally with 0.2 mg/kg of body weight for 4 weeks starting from 1st day p.i. ART: intramuscularly single dose of 300 mg/kg of body weight after 49 days p.i. PZQ: 500 mg/kg for 2 consecutive days	*N. sativa* as well as the combination of ART or PZQ resulted in significant increase in IL-2, IL-12 and TNF-α activities in *S. mansoni* infected mice as well as treatment of NS in non-infected. *N. sativa* in combination with ART or PZQ accelerate healing pathological granulomatous lesions of liver architecture and improved host immunity by stimulating cytokines.	[133]
	Antischistosomal activity and antioxidant effects of NS alone or combined with garlic extracts on experimentally *S. mansoni* infected mice.	Garlic extract 125 mg/kg p.i. and NS oil 0.2 mg/kg alone or combine for successive 28 days, starting 1st day p.i.	All treatment regimens significantly affected oogram pattern: treatment with compounds alone resulted in reduction of percentage of mature eggs while combine regimen resulted in increase of percentage of dead eggs. Administration of garlic extract prevent GSH depletion on infected mice. Combine regimen had more significant effect on serum enzymes (AST and ALP).	[134]
Curcumin	Assess curative effect of oil extract in liver cells of *S. mansoni* infected mice in compaison to PZQ	PZQ: 500 mg/kg by 2 consecutive days Extract: 300 mg/kg bw after one month p.i., twice a week for 2 months	Curcumin normalize the concentration of protein, glucose, AMP-deaminase and adenosine deaminase which were altered by infection Lowered pyruvate kinase level while PZQ induce more elevation; More potent rather PZQ in reducing egg count but no lowering worm burden. Most likely, antifecundity effect of curcumin might be involve in impairment or adult worms.	[135]
	Evaluation of schistosomicidal activity in vivo and immunomodulation of granulomatous inflammation and liver pathology in acute *S. mansoni* infection.	Total dose 400 mg/kg bw divided into 16 injections (2 injections per week for 8 consecutive weeks) starting from the first week of infection.	Effective in reducing worm (44.4%) and tissue-egg burdens; Reduction granuloma volume and liver collagen (79%); Restore hepatic enzymes activities to normal levels and enhanced catalase activity; Low serum level of both IL-12 and TNF-α; Augmented specific IgG and IgG1 responses against both SWAP and SEA.; It modulates cellular and humoral responses.	[136]
	Evaluation its role on induction of apoptosis and oxidative stress in couples of adult *S. mansoni* worms in vitro	1.56 to 100 μM incubated for 6, 12 or 24 h.	Significantly decreases the viability of adult female and male worms; Induce separation of couples and morphological alteration on mitochondria; Induce formation of SOD and increase its activity in adult worms; Alters several oxidative stress parameters in adult worms such decrease of GST, GR and GPX culminating in the oxidation of protein: Generates oxidative stress followed by an apoptotic-like event in adult worms, which ultimate leads to their dead.	[137]
β-carotene	Evaluation the protective effect on experimentally *S. mansoni* infected mice and on major enzymes activities involved in liver redox.	PZQ, 7 weeks p.i., 500 mg/kg (full dose) or PZQ ED_50_ 74.64 mg/kg βC, 2.7 mg/kg, 1 week before infection. βC + PZQ ED_50_ as mentioned	Produced significant reduction in worm burden (total number of worms: PZQ: 11.57 ± 0.59; PZQ (full dose): 0.46 ± 0.14; βC: 17.64 ± 1.11; βC + PZQ: 8.38 ± 0.51) accompanied with increase of dead ova and decrease in percentage of mature ova; reduced liver granuloma diameter. Combined regimen improved these parameters. Combined regimen improved the effect of antioxidant enzymes (as GPX and GST) and increase serum ALT and GGT. βC has protective effects against liver fibrosis which may be due to ability to encounter or minimize the formation of schistosomal products.	[138]
*N*-Acetyl-cysteine	Study immunopathological changes in murine schistosomiasis alone or in combination with PZQ.	NAC (200 mg/kg/day on 1st day after infection for acute phase; On 45th for the intermediate; 59 and 75th for chronic phases. PZQ (100 mg/kg) from 45th to 49th day p.i.	NAC alone did not present any schistosomicidal activity; animals treated with NAC and/or PZQ showed a reduction in the size of granulomas and those treated with NAC exhibited a lower degree of fibrosis. NAC functions as a direct scavenger of NO and peroxinitrite which are related to reductions of IFN-γ levels and increasing of IL-10 synthesis; Induce an immunomodulatory effect and reduce liver damage during granulomatous inflammation.	[139]
	Investigate ability of NAC to enhance potential of ART against adult *S. mansoni* worms and evaluates protective role on oxidative stress.	NAC-300 mg/kg 5 days a week for 4 weeks ART-300 mg/kg 7 weeks p.i. NAC + ART (as described)	Combine regimen approximately recovered levels of serum enzymes, content of GSH and activities. Decrease the total number of worms and hepatic ova count. ART alone produce valuable modulations in the hepatic activities; NAC may prevent experimental liver injury by modulating and enhancing GSH content and GSH-dependent antioxidant enzyme activities. Total worms: ART: 7.6 ± 1.5; NAC: 17.7 ± 1.5; NAC + ART: 3.3 ± 1.1.	[140]

PZQ: praziquantel; Resv-Resveratrol; Mel-melatonin; BDHQ: 4-hydroxy-quinolin-2(1H)-one; ART: artemether; NS: *Nigella sativa*; βC: β-carotene; NAC: *N*-acetyl-cysteine; AS: aspidin; FAA: flavaspidic acid; MbA: methylene-*bis*-aspidinol; DA: desaspidin; Syl: sylimarin; p.i.: post-infection bw: body weight; GSH: glutathione; GR: glutathione reductase; SOD: superoxide dismutase; GGT: gamma-glutamyl transferase; CAT: catalase; TrxR: thioredoxin reductase; SDH: succinate dehydrogenase; GPx: glutathione peroxidase; HK: hexokinase; G-6-PDH: glucose-6-phosphate dehydrogenase; DPHH: 1,1-diphenyl-2-picrylhydrazyl; NOx: nitrogen oxide; IgG: immunoglobin G; IgE: immunoglobin E; IgM: immunoglobin M; IL-2: interleukin-2; IL-10: interleukin-10; IL-12: interleukin-12; IFN-γ: interferon gamma; TNF-α: tumor necrosis factor α; ICAM-1: intracellular adhesion molecule; ALA: alanine aminotransferase; AST: aspartate transaminase; MDA: malondialdehyde; LPD: lipoamide dehydrogenase; LDH: lactate dehydrogenase; ALP: alkaline phosphatase; AMP: adenosine monophosphate; SEA: soluble egg antigen; CAP: cercarial antigen preparation; SWAP: soluble worm antigen preparation.
